# Second hand smoke exposure in public venues and mental disorder: a representative nationwide study of China

**DOI:** 10.1186/s12971-015-0046-7

**Published:** 2015-07-17

**Authors:** Tingzhong Yang, Chengjian Cao, Randall R. Cottrell, Dan Wu, Lingwei Yu, Haoxiang Lin, Shuhan Jiang, Kathleen J. Young

**Affiliations:** Center for Tobacco Control Research, Zhejiang University School of Medicine, Yuhangtang Road, Hangzhou, 310058 China; Hangzhou Hospital for the Prevention and Treatment of Occupational Diseases, Hangzhou, 310021 China; Public Health Studies Program, School of Health and Applied Human Sciences, University of North Carolina, Wilmington, NC 28403 USA; Tobacco Control Office, Chinese Center for Disease Control and Prevention, Beijing, 100050 China; Department of Health Sciences, California State University, Northridge, CA 91330 USA

**Keywords:** Secondhand smoke, Mental disorders, SHS control policy, Public education, China

## Abstract

**Introduction:**

Many studies have clearly linked exposure to Second Hand Smoke (SHS) to various somatic diseases, however, few studies have examined the relationship between SHS and mental disorders and those that have were only conducted with specific groups. The purpose of this study was to examine the association between SHS exposure and mental disorders among Chinese residents in both worksites and public places.

**Methods:**

A cross-sectional multistage sampling design was used to interview subjects from 21 selected cities in China. Using a standardized questionnaire including demographic characteristics, SHS exposure, and mental health information was collected. Multiple logistic regression analysis was used to assess the association between SHS exposure and mental disorders.

**Results:**

Sixteen-thousand-eight-hundred-sixty-six valid questionnaires were collected and utilized in this study. Of 11,206 non-smokers, SHS exposure prevalence in workplaces and public places were respectively 78.4 % (95 % C.I.:74.3–82.5 %) and 80.7 % (95 % C.I.:74.6–86.5 %). Multiple logistic regressions showed SHS exposures in these venues were positively related to mental disorder.

**Conclusions:**

These findings further support the health hazards of SHS exposure. Findings underscore the importance of controlling SHS exposure, and can be used to inform future SHS control policies and reinforce the need for public education in China.

## Introduction

Secondhand smoke (SHS), also known as environmental tobacco smoke (ETS) or tobacco smoke pollution (TSP) or passive smoke, is formed from the burning of cigarettes and other tobacco products and from smoke exhaled by smokers [38]. The World Health Organization (WHO) has estimated that in 2004, about one-third of adults and 40 % of children worldwide were exposed to SHS [38]. The International Labor Organization (ILO) estimates that at least 200,000 workers die every year from exposure to secondhand tobacco smoke [35]. Currently 740 million Chinese people are exposed to SHS, including 180 million children under the age of 15 [19]. Several studies found SHS was very common in households, workplaces and public places [33, 41, 46].

A mental illness is a condition that impacts a person’s thinking, feeling or mood may affect and his or her ability to relate to others and function on a daily basis [23]. Many individual and environmental factors influence mental disorders. Some studies have clearly linked exposure to SHS to various somatic diseases [6, 7, 15, 34, 36], however, few studies have examined the relationship between SHS and mental disorders [1, 2, 11, 22]. Furthermore, there have been no studies reporting on this relationship in China. The purpose of the present study was to explore the possible association between SHS exposure in work and public places and mental disorders in a population-based sample of Chinese adults.

## Methods

### Study area and participants

This study employed a cross-sectional multistage sampling design. In Stage 1, 21 cities, with over 1.5 million in population, were purposefully selected from across China and differentiated by regional location: Nine were located in eastern China, five in central China, and the remaining seven in the west. About 68 % of Chinese provinces were covered by the 21 cities in this study. Most of the sample cities were provincial capitals, but we included a few non-provincial capital cities because of their size (over 1.5 million population) in order to achieve better regional representation. Stage 2 involved selection of residential districts within each city. Two residential districts were randomly selected in the main urban zone of each city. This study included urban residents, the main urban zones were those areas where most permanent residents live, excluding new building districts and sub-districts where many rural migrants reside. In Stage 3, four communities were randomly selected within each residential district. In Stage 4, a family household registration (“hukou”) list was used to randomly sample households in each community. Individuals aged 15 years and older, who had lived in their home for at least a year, were identified within each household [5, 40–42, 46]. Finally, one eligible participant was randomly selected from each family, with eligibility being determined by birthdate closest to the contact date [46].

### Data collection

Smoking, SHS exposure in Public Venues, mental status, and other variables were assessed through a self-administered questionnaire. The questionnaire was personally delivered to each participant by a trained field staff member who also answered questions, collected the completed questionnaires and reviewed them to make sure they were complete. This is the conventional data collection procedure for population-based tobacco control surveys such as the GATS tobacco control survey [5, 40]. The appropriateness of this data collection process is reinforced by evidence that self-report bias in smoking research is minimal [14, 29, 37]. Since smoking represents normative behavior for adults in China, any social expectation of accurate reporting about smoking and related information is plausible but a minor concern [42]. Field staff members were fourth-year medical students from local medical colleges, who had received a 1-day training on study protocol and interviewing procedures. The questionnaire was administered privately in participants’ homes or in a designated quiet place, such as a backyard or community park. Survey were conducted on Saturdays, Sundays, weekday evenings or other times when participants were available. After receiving oral instructions by staff about the survey and questionnaire, the questionnaire was given to each participant to complete. Completion took approximately 30 min. Participants could ask staff if they were confused about any items on the questionnaire. For participants with low educational levels or physical limitations, a survey staff member read the entire questionnaire. Each participant was given an opportunity to clarify confusing questions and adequate time to complete the questionnaire. Participants were requested to resolve any omissions, as appropriate, and were given a token of appreciation (toothbrush and toothpaste, or other small gifts valued at approximately US$ 1.00) for their time.

The same research protocol was utilized across all 21 cities to assure homogeneity of interview and data collection. The survey protocol including the use of small gifts and the questionnaire was approved by the Ethics Committee at the Medical Center, Zhejiang University, and verbal consent was obtained from all participants or their guardian prior to the commencement of the study. Our methods have been extensively employed in smoking research in China, and they possess acceptable validity [5, 46].

### Measures

A current smoker was defined as someone who smoked cigarettes at the time of the interview. Smokers were excluded from the study. Only non-smokers participated in this study.

Sociodemographic information was collected on the variables of: age, gender, ethnicity, educational level, occupation, person income, and regional location. Person income was estimated as the average income per person in a household during the prior year.

For the purpose of this study SHS exposure included exposure in public venues such as workplaces and public places [23, 46]. With regard to workplaces respondents were asked how often they were exposed to SHS in all indoor work areas. Possible responses included: never, rarely, sometimes, often, and always. For retired or unemployed respondents, “workplace” referred to the places where they spent much of their time such as part-time employment sites, and places where they may participate in leisure activities in their community or elsewhere. For students, “workplace” covered such places as classrooms and libraries [46]. Concerning SHS exposure in public places, participants were asked two questions: (1) “Have you been in restaurants, hospitals, shops, buses, and other public places in the past 6 months?” (yes/no), and (2) If “yes”, they were asked how often they were exposed to SHS in these facilities. Response options were never, rarely, sometimes, often, and always [5, 43, 46].

Mental health status was measured by the Chinese Health Questionnaire (CHQ), which was derived from the General Health Questionnaire (GHQ), and has been used to screen for mental disorders in community settings [8, 45]. This questionnaire has been widely used to assess mental disorders in China and has been shown to be an appropriate indicator of mental health status [12, 18, 27, 45]. The CHQ is a self-administered, 12-item instrument designed for detecting mental disorders in the community and among primary health care patients. It has acceptable reliability and validity and is commonly utilized for research purposes in community and primary care settings in China [12, 18, 27]. It has a four-point scale for responses: “not at all” and “same as usual” both = 0 and “rather more than usual” and “much more than usual” = 1. The total score, obtained by summing up the scores for the individual items can range from 0 to 12, and measures the severity of mental disorders. A cut-off score of 3 or more on the CHQ was classified as a higher score, which signified mental disorders [1, 45].

### Analysis

All data were entered into a database using Microsoft Excel. The dataset was then imported into SAS (9.3 version) for the statistical analyses using the SAS 9.3 survey procedure [32]. Descriptive statistics were calculated for SHS exposure status. Both unadjusted and adjusted logistic regression methods were considered in the analyses. The unadjusted method used only the key factors of interest as independent variables in the analyses, while the adjusted method added all of the possible confounders listed in Tables 1 and 2 as covariates in the logistic models. For this analysis, we operationalized our dependent variable, mental disorder, as a binary response (no = 1, yes = 0). The independent variables in this analysis were those emerging as statistically significant in the unadjusted method. All categorical, they are listed in Table 2. The first category in each variable served as the referent in the multiple logistic regression analysis. Backward stepwise regression is a preferred method for exploratory analyses, where analysis begins with a full or saturated model and variables are eliminated from the model in an iterative process. *P*-values less than or equal to 0.05 indicated statistically significant differences.

**Table 1:** Demographic characteristics of the urban sample China, 2011

Group	Number	% of sample
Age (years)		
< 25	2110	15.0
25–34	3061	20.8
35–44	2537	19.4
45–54	1620	20.3
55+	1878	25.5
Gender		
Male	3747	28.6
Female	7459	78.8
Ethnicity		
Han	9776	89.7
Other	1430	10.3
Education		
Elementary school or less	634	5.3
Junior high school	2390	18.3
High school	4089	37.4
Junior college or more	4093	39.0
Occupation		
Managers and clerks	576	4.2
Professionals	580	4.1
Commerce and service	1254	10.0
Operations	2811	22.5
Students	1841	24.7
Retired	1319	8.5
Other	2825	25.9
Income/person/year (Yuan)		
< 10,000	1416	12.9
10,000–19,999	3130	24.8
20,000–29,999	3103	26.1
30,000–39,999	1413	14.6
40,000–49,999	990	10.2
50,000+	1154	11.5
Region location		
East	4556	51.6
Middle	1664	9.9
West	4986	38.6

**Table 2:** Mental disorder prevalence among urban residents in China, 2011

Group	Number	Mental disorder prevalence (%)	Unadjusted OR
Age (years)			
< 25	2110	22.7	1.00
25–34	3061	23.2	1.03 (0.88,1.21)
35–44	2537	19.3	0.82 (0.64,1.04)
45–54	1620	21.0	0.91 (0.73,1.12)
55+	1878	20.3	0.87 (0.61,1.26)
Gender			
Male	3747	14.1	1.00
Female	7459	24.1	1.93 (1.07,3.46)**
Ethnicity			
Han	9776	17.9	1.00
Other	1430	50.6	4.67 (2.12,9.44)
Education			
Elementary school or less	634	23.2	1.00
Junior high school	2390	13.7	0.52 (0.30,0.92)**
High school	4089	7.9	0.28 (0.13,0.64)***
Junior college or more	4093	37.2	1.96 (1.15.3.33)***
Occupation			
Managers and clerks	576	21.7	1.00
Professionals	580	19.6	0.88 (0.30,0.92)
Commerce and service	1254	19.3	0.87 (0.55,1.37)
Operations	2811	10.3	0.42 (0.23,0.76)***
Students	1841	4.5	0.17 (0.05,0.55)***
Retired	1319	42.6	2.69 (2.69,5.19)***
Other	2825	40.5	2.16 (1.26,4.82)***
Income/person/year (Yuan)			
< 10,000	1416	23.3	1.00
10,000–19,999	3130	12.1	0.45 (0.26,0.80)***
20,000–29,999	3103	8.6	0.31 (0.14,0.69)***
30,000–39,999	1413	14.1	0.54 (0.18,1.56)
40,000–49,999	990	39.4	2.16 (0.97,4.82)*
50,000+	1154	60.3	5.01 (0.41,17.76)**
Region location			
East	4556	19.6	1.00
Middle	1664	21.8	1.14 (0.74,1.77)
West	4986	23.2	1.23 (0.81,1.88)
SHS exposure in work place			
Never	530	18.1	1.00
Rarely	1869	11.1	0.56 (0.26,1.21)
Sometime	5357	11.4	0.58 (0.31,1.09)*
Often	2230	30.1	1.95 (0.98,3.87)*
Always	1220	71.3	11.25 (3.90,32.45)***
SHS exposure in public place			
Never	365	19.5	1.00
Rarely	1729	14.9	0.72 (0.40,1.31)
Sometime	5629	9.0	0.41 (0.21,0.81)**
Often	1730	39.2	2.66 (1.29,5.48)***
Always	554	61.6	6.61 (2.40,18.20)***

*P < =0.01, **P < =0.05, ***P < =0.005

All analysis were weighted [3, 10]. Weights included (1) sampling weights as the inverse of the probability of selection, which were calculated at regional, city, district, and community levels, and they were multiplied together for each level. We did not consider households in our weighting, since family size was rather uniform given population policy and family planning (approximately 3 people per household); thus, the probability of participant selection was essentially the same across households. (2) The non-participation weight covered household and individual aspects. Household-level non-participation weights were calculated at the city level. Individual-level non-participation weights were based on a combination of city, age, and sex, and the corresponding weight was the inverse of the participation rate. The overall non-participation weight was the product of the household and individual weights (3) post-stratification weights reflected a combination of sex (male, female) and age (<25 year, 25–34, 35–44, 45–54, and 55+) based on estimated distributions of these characteristics from a national survey [24]. The final overall weights were computed as the product of the above three weights.

## Results

A total of 18,875 individuals were identified as potential subjects for this study, among whom, 17,124 were effectively contacted and agreed to participate in the survey. Of the 17,124 surveys collected, 16,866 (98.49 %) were complete and valid questionnaires utilized in this study. Of the respondents (*n* = 16,866), 11,206 were non-smokers and were thus included in this study. Of 11,206 participants, 79 % of them were female. Sixty-one percent had high school or less education, more participants were engaged in occupations of operations, students, and commerce and service (Table 1).

The mental disorder prevalence varied across age, education, occupation, income, and SHS exposure prevalence in workplaces and public places (Table 2).

Table 3 showed that the mental disorder prevalence was higher in females than males, in elementary school or less than junior high school and high school, and in <10,000 income as compared to those in the 10,000–40,000 group.

**Table 3:** Multiple logistic regression analysis of SHS exposure on mental disorder in China, 2011

Group	Number	Mental disorder prevalence (%)	Adjusted OR
Gender			
Male	3747	14.1	1.00
Female	7459	24.1	1.50 (1.04,2.15)**
Education			
Elementary school or less	634	23.2	1.00
Junior high school	2390	13.7	0.64 (0.46,0.88***
High school	4089	7.9	0.42 (0.24,0.73)***
Junior college or more	4093	37.2	1.12 (0.72,1.76)
Income/person/year (Yuan)			
< 10,000	1416	23.3	1.00
10,000–19,999	3130	12.1	0.49 (0.33,0.73)***
20,000–29,999	3103	8.6	0.33 (0.18,0.59)***
30,000–39,999	1413	14.1	0.39 (0.17,0.91)**
40,000–49,999	990	39.4	1.18 (0.57,2.52)
50,000+	1154	60.3	1.30 (0.58,2.95)
SHS exposure in work place			
Never	530	18.1	1.00
Rarely	1869	11.1	0.70 (0.48,1.02)
Sometime	5357	11.4	0.83 (0.58,1.19)
Often	2230	30.1	0.98 (0.64,1.50)
Always	1220	71.3	2.24 (1.23,4.08)***
SHS exposure in public place			
Never	365	19.5	1.00
Rarely	1729	14.9	0.94 (0.60,1.45)
Sometime	5629	9.0	0.56 (0.30,1.03)
Often	1730	39.2	1.74 (0.91–3.35)
Always	554	61.6	2.14 (1.19–3.87)**

*P < =0.01, **P < =0.05, ***P < =0.005

Those persons reporting SHS exposure prevalence in workplaces and public places were respectively 78.4 % (95 % C.I.:74.3–82.5 %) and 80.7 % (95 % C.I.:74.6–86.5 %). The unadjusted and adjusted logistic regressions analysis showed that SHS exposures in these venues were positively related with mental disorders (Tables 2 and 3).

## Discussion

The purpose of this study was to examine the association between SHS exposure and mental disorders among Chinese residents in both worksites and public places. Results found Secondhand smoke exposure was positively associated with mental disorders among nonsmokers. Our results are consistent with data from previous studies and suggest that exposure to SHS may precipitate the onset of or exacerbate mental disorder symptoms [1, 2, 11, 22].

### SHS–mental health association

The mechanism by which SHS exposure may precipitate poor mental health is unclear. Several possible explanations may account for this association. SHS exposure may be a proxy to stressful living and working environments, and stress has been associated with mental disorders [10, 13]. Indeed, the Chinese culture is strongly influenced by Confucianism which emphasizes the notions of “ren”,” yi”, and “li”. People do not directly express their feelings when dealing with troubles, most will refrain from saying no in order not to lose “face” when confronted with the intrusion of SHS. Instead, they always endure to the extent of harming mental health. Those exposed to SHS may feel frustrated that they cannot express their feelings or do anything to change the unpleasant environment.

There are many types of events or situations that generate stress, including SHS exposure. The outcome resulting from such stress include poor mental health and well being, to mental illness and mental disorders [26]. Animal data have indicated that tobacco can induce negative moods [21], suggesting that tobacco exposure may be a direct cause of psychiatric illness. Further, other biological mechanisms might include low-grade inflammation, which is elevated with SHS exposure and associated with mood disorders such as depression [28]. Petty et al. found that Chronic exposure to cigarette smoke may lead to lower levels of dopamine and r-aminobutyric acid (GABA), which are associated with mood disorders [25]. Another possible explanation is that SHS exposure is associated with chronic diseases [6, 7, 15, 34, 36], and chronic diseases may be associated with mental disorders. Taken together, evidence indicates a possible causal role of SHS exposure in mental health. The current study, however, utilized a cross-sectional design; therefore, cause and effect cannot be established. Since passive smoking is not an active behavior, it is unlikely that an effect in the opposite direction, (i.e., poor mental health leading to passive smoking) could logically take place. Furthermore, this study included a large, representative, general population–based sample, and findings met several criteria for the inference of causality, including strength of some of the associations, consistency of the association, and the plausibility of the effect, which indicate a high possibility of causal inferences.

### Frequency of SHS Exposure

This study also confirmed SHS exposures in Chinese public venues were very common, with exposure prevalence in workplaces and public places respectively 78.4 and 80.7 %. A prior study also reported that exposure to tobacco smoke in public places was common, in most restaurants and 70 % of schools, hospitals, government buildings, and train stations [33]. Another study suggested SHS exposure prevalence was 63.3 % in work places [41]. SHS pollution is a severe public health problem in China.

### China smoking free policy

According to the World Health Organization [39] smoke free policies are the most effective way to reduce exposure to tobacco smoke in public venues. In order to combat the global spread of tobacco use and secondhand tobacco smoke exposure, the WHO established the Framework Convention on Tobacco Control (FCTC) in 1999. In ratifying the FCTC, the Chinese government agreed that all workplaces and public places should be smoke free by 2011. To meet this objective, efforts were made to expand the number of smoke free places throughout the country. Although China lacks a comprehensive smoke free law, several national laws and policies regulate smoking in public places. On May 1, 2011, the Chinese Ministry of Health released “*Guidelines on the Regulatory Measures of the Sanitary Administration in Public Places*,” an action which indeed strengthened control measures on secondhand smoke in public places [17, 20]. Nearly half of the Chinese midsize and large cities had instituted smoke free regulations in public places by the end of 2010 [17]. Despite these control measures and regulations SHS exposure in China were still high. This may be due to (1) some policies or regulations did not meet the requirements of the Framework Convention on Tobacco Control, which covered all public places and all indoor spaces, (2) the regulation implementation and enforcement was weak, (3) there has been a lack of public education concerning the harmful effects of SHS [17, 20, 43]. Efforts to restrict smoking in public places in China should strongly promote the new policies and regulations and strengthen the implementation and enforcement process, while simultaneously raising public awareness of the perils of secondhand smoke.

### Tobacco program implications

The findings presented in here provide new evidence on the harmful effects of SHS exposure on mental health among this representative sample of Chinese residents. Studies have shown that banning smoking in workplaces and other public settings leads to immediate reductions in mental specific symptoms [9, 16, 31]. Both SHS exposure and poor mental health are major public health problems. Smokefree policies are the most effective way to reduce exposure to tobacco smoke in public venues, and also have a positive influence on smoking in households [44]. Public education campaigns need to promote greater awareness of the negative consequences associated with SHS exposure and reinforce the importance of laws that restrict smoking. These campaigns should be required by the government and be implemented in public schools, universities, hospitals, worksites, and community agencies. In addition multimedia campaigns involving television, radio, billboards, newsprint, and transit messaging should be initiated to augment the site specific interventions.

### Study limitations

Several limitations to our study must be considered when interpreting the results. As mentioned above the cross-sectional study design is an important limitation of our study, therefore, a causal link between SHS exposure and mental disorders cannot be established with this work. As mentioned, however, a relationship between SHS and mental disorders does exist and since it is unlikely that mental disorders lead to SHS exposure, the likelihood that SHS exposure leads to mental disorders is increased. SHS exposure as measured in this study is subjective and self-reported; therefore, it is possible that the amount of SHS exposure could be over or under reported. For example, those who have a strong aversion to SHS or are very concerned and aware of their health may overestimate exposure to SHS; whereas, some people may underestimate exposure to SHS due to lack of concern or misinformation about the health impact of SHS. However, self-reported data are the conventional data collection procedure for population-based tobacco control surveys, including the GATS tobacco control survey [5, 40]. Many studies have noted that self-report bias in smoking research is minimal [14, 29, 37]. A more precise and objective gauge of SHS exposure is PM2.5 measurement, but this method is not practical in population surveys. Recall bias could also be a concern, but considering the timeframe used in this study is the recent past recall bias should be limited [7].

In this study participants were only asked to report on SHS exposure in workplaces and public places, and did not include SHS exposure in other settings. It is possible that smoking restrictions in various venues may be related to SHS exposure [4, 30, 47]. It is also possible that exposure to SHS in other venues are in someway related to SHS in workplaces and public places and may be a confounding factor in assessing the relationship between SHS exposure in workplaces and public places and mental disorders. However, it matters little whether SHS exposure in workplaces and public places or SHS exposure in other venues are associated with mental disorders, from a public health perspective, they have the same significance. Finally, one other limitation may be some potential confounders that were not controlled for in this study, such as, subjects’ disease history.

## Conclusion

Based on the results of this study exposure to SHS is common among the study participants and there is a significant relationship between SHS and mental disorders. This study provides new evidence on the mental health hazards of SHS exposure in public venues and worksites among Chinese urban residents. These findings underscore the importance of controlling SHS exposure, and can be used to inform future SHS control policies and reinforce the need for public education in China.
